# A collection of novel *Lotus japonicus LORE1* mutants perturbed in the nodulation program induced by the *Agrobacterium pusense* strain IRBG74

**DOI:** 10.3389/fpls.2023.1326766

**Published:** 2024-01-05

**Authors:** Ivette García-Soto, Stig U. Andersen, Elizabeth Monroy-Morales, Mariana Robledo-Gamboa, Jesús Guadarrama, Norma Yaniri Aviles-Baltazar, Mario Serrano, Jens Stougaard, Jesús Montiel

**Affiliations:** ^1^ Centro de Ciencias Genómicas, Universidad Nacional Autónoma de México (UNAM), Cuernavaca, Mexico; ^2^ Department of Molecular Biology and Genetics, Aarhus University, Aarhus, Denmark

**Keywords:** symbiosis, legume (nodules), mutant screening, autophagy, intercellular infection, *Lotus japonicus*

## Abstract

The *Lotus japonicus* population carrying new *Lotus* retrotransposon 1 (*LORE1*) insertions represents a valuable biological resource for genetic research. New insertions were generated by activation of the endogenous retroelement *LORE1a* in the germline of the G329-3 plant line and arranged in a 2-D system for reverse genetics. *LORE1* mutants identified in this collection contributes substantially to characterize candidate genes involved in symbiotic association of *L. japonicus* with its cognate symbiont, the nitrogen-fixing bacteria *Mesorhizobium loti* that infects root nodules intracellularly. In this study we aimed to identify novel players in the poorly explored intercellular infection induced by *Agrobacterium pusense* IRBG74 sp. For this purpose, a forward screen of > 200,000 *LORE1* seedlings, obtained from bulk propagation of G329-3 plants, inoculated with IRBG74 was performed. Plants with perturbed nodulation were scored and the offspring were further tested on plates to confirm the symbiotic phenotype. A total of 110 *Lotus* mutants with impaired nodulation after inoculation with IRBG74 were obtained. A comparative analysis of nodulation kinetics in a subset of 20 mutants showed that most of the lines were predominantly affected in nodulation by IRBG74. Interestingly, additional defects in the main root growth were observed in some mutant lines. Sequencing of *LORE1* flanking regions in 47 mutants revealed that 92 *Lotus* genes were disrupted by novel *LORE1* insertions in these lines. In the IM-S34 mutant, one of the insertions was located in the 5´UTR of the LotjaGi5g1v0179800 gene, which encodes the AUTOPHAGY9 protein. Additional mutant alleles, named *atg9*-2 and *atg9*-3, were obtained in the reverse genetic collection. Nodule formation was significantly reduced in these mutant alleles after *M. loti* and IRBG74 inoculation, confirming the effectiveness of the mutant screening. This study describes an effective forward genetic approach to obtain novel mutants in *Lotus* with a phenotype of interest and to identify the causative gene(s).

## Introduction

Plant mutant collections have become a key resource to perform functional genomics and characterization of genes-of-interest in various biological processes. Mutagenized populations can be obtained with physical, chemical or biological mutagens (Chaudhary et al., 2019). The availability of mutant collections in the model legumes *Medicago truncatula* and *Lotus japonicus* (*Lotus*) have contributed importantly to understand the genetic networks that govern legume-rhizobia symbiosis (Roy et al., 2020). In the *M. truncatula* R108 line, a large mutant population was generated with the tobacco retrotransposon element Tnt1 (Tadege et al., 2008; Pislariu et al., 2012). In the *Lotus* accession Gifu, genotyping of plants regenerated from tissue culture (Madsen et al., 2005), identified new insertions of an endogenous *LORE1* retrotransposon and further investigation showed epigenetic activation of the *LORE1a* element in the germline (Fukai et al., 2008; Fukai et al., 2010). Subsequent studies exploited this discovery, generating a reverse genetic mutant collection of 134,682 individual plants from the G329-3 line carrying an active *LORE1a*. In this collection, information of the insertion sites was obtained by sequencing, facilitating identification and characterization of mutants disrupted in genes-of-interest (Fukai et al., 2012; Urbanski et al., 2012; Malolepszy et al., 2016).

*Lotus* establishes a mutualistic association with its cognate symbiont *Mesorhizobium loti*. The symbiotic association occurs in the rhizosphere, where both organisms exchange chemical signals for the specific recognition. This compatibility activates a symbiotic signaling pathway that allows intracellular rhizobial infection via root-hair infection threads (ITs) and initiation of the nodule organogenesis program. Bacteria migrate through the ITs towards the dividing cortical cells in the nodule primordia, that give rise to a nitrogen-fixing nodule. In the nodule cells, rhizobia are encapsuled into membranous structures called symbiosomes, where they convert atmospheric nitrogen into ammonia (Downie, 2014). *Lotus* is able to establish symbiotic associations with a broad spectrum of rhizobial species (Gossmann et al., 2012; Sandal et al., 2012; Zarrabian et al., 2022), and it was recently shown that is effectively nodulated by IRBG74, an *Agrobacterium pusense* strain isolated from *Sesbania cannabina* nodules (Cummings et al., 2009). IRBG74 induces massive root hair curling in *Lotus*, followed by intercellular infection of the epidermal cells (Montiel et al., 2021). Intercellular colonization is an entry mode observed in approximately 25% of the legume species investigated and although this group includes major legume crops, genetic control remains largely unexplored (Sprent, 2007). However, recent discoveries in intercellularly infected legumes such as *Arachys hypogaea*, *Aeschynomene evenia* and *Lotus*, have attracted attention to this entry mode and provided valuable information about the process (Chaintreuil et al., 2016; Peng et al., 2017; Bertioli et al., 2019; Chen et al., 2019; Karmakar et al., 2019; Montiel et al., 2021; Quilbe et al., 2021; Raul et al., 2022; Montiel et al., 2023). While intra- and intercellular rhizobial invasion share some genetic components and transcriptional responses, relevant differences were found among these processes (Quilbe et al., 2022).

*Lotus* is an optimal organism with abundant resources to analyze different plant-microbe interactions, such as germline activation of endogenous *LORE1*a retrotransposon, where new insertion sites can be tracked in the genome with a relatively simple and quick sequence-based method (Urbanski et al., 2012). Here we assess the potential use of a *LORE1a* activated population in a bulk forward screening. We present a collection of novel mutants impaired in intercellular symbiotic colonization by IRBG74 and compare the nodulation phenotype with that obtained by intracellular *M. loti* colonization. The genome mapping of the *LORE1* insertion sites allowed us to identify *ATG9* as a novel regulator in *Lotus-*rhizobia symbioses.

## Materials and methods

### Germination of *LORE1* seeds and mutant screening

Seeds from the line G329-3 were collected during several harvesting periods to obtain a mixed and balanced population. Batches with thousands of *LORE1* seeds were scarified with hydrochloric acid for 20 min, followed by several washes with distilled sterile water. The seeds were placed in square Petri dishes with moistened paper and two days later, transferred to autoclaved square boxes (40x40x40 cm) filled with Leca. The swollen seeds were inoculated with IRBG74 (O.D. 0.05) and the boxes were maintained in a growth room with controlled temperature, photoperiod (21° C; 16:8 h), intensity light (100 µmol/m2/s). The plants were watered once per week with B & D solution (Broughton and Dilworth, 1971). This procedure was repeated for several months until approximately 200,000 plants were screened. We selected mutants with a visible nodulation phenotype such as: Nod-, hypernodulation or Fix-. This latter manifested by white nodules, reduced nodulation, short plant size and yellow leaves. The selected lines were kept at the greenhouse for seed production. Ten offspring plants from each mutant were subsequently tested by a nodulation assay in plate with IRBG74 (O. D. 0.05).

### Identification of *LORE1* elements

To track the *LORE1* elements in the mutant collection, we followed the protocol previously described by Urbanski et al. (2012). First, total DNA was isolated from 3-5 plant of each mutant with hexadecyltrimethylammonium bromide (CTAB) (Rogers and Bendich, 1985). The DNA was quantified and shared with a Covaris S-series instrument to obtain 600-800 bp fragments. The DNA was blunted, end-repaired and adenylated with a T4 DNA polymerase, T4 DNA polynucleotide kinase and Taq polymerase, respectively, following the manufacturer´s instructions. A ligation was performed to incorporate a splinkerette intermediate adaptor (IA) to the DNA sequences (Mikkers et al., 2002), using a T4 DNA ligase. The flanking *LORE1* fragments were obtained by sequential PCR reactions. The first PCR products were amplified with the Splink1 and P2 oligonucleotides, obtaining amplicons of 500-600 bp, that were excised from the agarose gels and used as template for a nested PCR using the Splink2 and P3 primers (**Supplementary Table S2**). For each DNA sample, a unique P3 oligonucleotide harboring a molecular barcode was used. With this procedure, PCR products of 200-400 bp were amplified and pooled at equimolar concentrations for Illumina sequencing (**Supplementary Table S2**). The sequencing data was processed to detect the *LORE1*, adaptor and barcode sequences. These regions were trimmed and only the genomic region was mapped to the *L. japonicus* Gifu genome using bowtie2 with the following parameters: –end-to-end -X 500 -N 1 -L 28 -D 20 (Kamal et al., 2020). Only insertion with ≥ 4 reads were considered for the analysis.

### Root growth and nodulation kinetics of *LORE1* mutants

The *L. japonicus* accession Gifu (Handberg and Stougaard, 1992) and *LORE1* lines were germinated as described above. For nodulation tests, seedlings of 3-5 days post-germination (dpg) were transferred to 12x12 cm square Petri dishes (10 seedlings per plate) containing 1.4% (w/v) agar slant with ¼ B & D medium (Broughton and Dilworth, 1971) and inoculated with 1 ml per plate of a bacterial suspension (*M. loti* R7A or IRBG74; OD600 = 0.05). The plants were kept in a growth room at 21° C with photoperiod (16/8 h). The nodule numbers were recorded weekly using a stereomicroscope. For the root growth dynamics, instead of B & D solution, the agar was supplemented with Gamborg’s B-5 basal medium (Sigma-Aldrich, G5893) and the progression of the apical main root was monitored weekly.

### Genotyping of *LORE1* lines

PCR reactions were performed to genotype the Nod- lines IM-A39, IM-N10 and IM-D22 using specific oligonucleotides for symbiotic genes (**Supplementary Table S2**), with the Phire Plant Direct PCR Mix (Thermofisher), following the manufacturer’s instructions. Nested PCRs were done for *NFR5* in the IM-A39 mutant to flank the *LORE2* insertion and the PCR product was sequenced. The *LORE1* lines, 30164676 (*atg9*-2) and 30077209 (*atg9*-3), affected in the LotjaGi5g1v0179800 gene (*LjATG9*) were genotyped according to the *Lotus* base guidelines (Mun et al., 2016).

## Results

### Novel *LORE1* mutants affected in the *Lotus*-IRBG74 association

**Figure 1:**
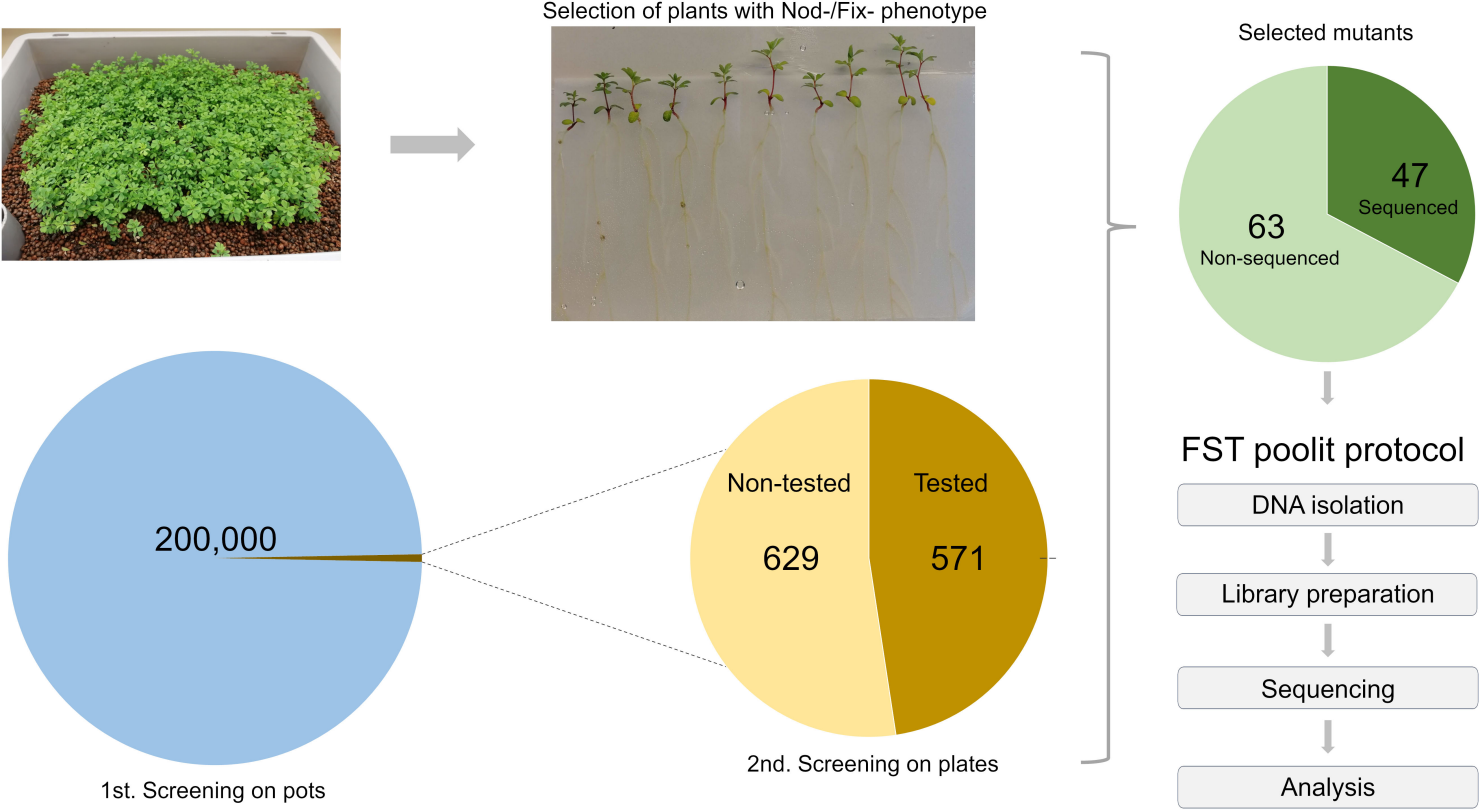
Scheme of the *LORE1* mutant screening to identify lines affected in the intercellular infection by IRBG74. Approximately 200,000 *LORE1* plants were inoculated with IRBG74, selecting 1,200 mutants with a Nod- or Fix- phenotype at 6-8 wpi. The offspring seeds of 571 selected lines were inoculated on plates (2^nd^ screening), confirming the nodulation phenotype in 110 lines. For this study, we conducted the FST poolit method to identify the *LORE1* insertions sites in the genome of 47 selected mutants.

Progeny of the G329-3 line was successfully used to generate the *LORE1* reverse genetics mutant collection in *Lotus*, composed of 134,682 individual plants (Urbanski et al., 2012; Malolepszy et al., 2016). To explore the potential of *LORE1* for forward genetics, cuttings of the G329-3 line were cultivated and harvested in bulk. In this study, several batches of these seeds were used in a large-scale forward screen, searching for mutant plants perturbed in the symbiotic association with IRBG74, a *Rhizobium* sp. that induce nitrogen-fixing nodules in *Lotus* through intercellular infection (Montiel et al., 2021). More than 200,000 *LORE1* seedlings were inoculated in boxes with IRBG74 and plants with aberrant nodulation phenotypes at 6-8 weeks post-inoculation (wpi) were screened out. Focusing especially on Fix- mutants and delayed nodulation likely to carry mutations in genes required for intercellular infection, 1,200 putative mutants were selected and transferred to the greenhouse for seed production. Large-scale mutant screenings tend to have a variable proportion of false positive selection, therefore, to confirm the symbiotic phenotype of the selected lines, ten offspring plants from each mutant were inoculated with IRBG74 in plates (**Figure 1**). Since this is an ongoing project, the nodulation phenotype of the progeny of 629 mutants, selected in the first screening still needs to be validated. However, in the remaining 571 lines, the plate test confirmed the nodulation phenotype in 110 mutants (**Figure 1**, **Supplementary Figure S1**). In this mutant population, 105 lines showed a Fix- phenotype manifested in white nodule appearance, however, we also identified 4 Nod- mutants without visible nodules and 1 hypernodulating mutant (**Supplementary Figure S1**).

### Differential symbiotic performance of *LORE1* mutants with *M. loti* and IRBG74

**Figure 2:**
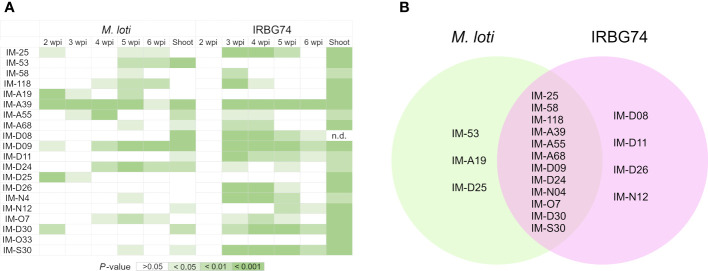
Significant reductions of nodule number and shoot length in *LORE1* mutants. **(A)** Graphical summary represented by shades of green of the significant differences observed by Mann–Whitney U-test for the parameters recorded in **Supplementary Figures S2A-F**. **(B)** Venn diagrams of the *LORE1* lines perturbed in the nodule formation induced by *M. loti* (green) and IRBG74 (pink).

In this work, we identified 110 *LORE1* mutants affected in the nodulation performance with IRBG74. Since handling this number of mutant lines represents a technical challenge, we focused our attention on a subset of 20 randomly chosen mutants. First, we explored the specificity of the symbiotic phenotype, by analyzing the nodulation kinetics on plates at 2-6 weeks post-inoculation (wpi) with *M. loti* and IRBG74 (**Supplementary Figures S2A-E**), which colonize *Lotus* roots intra- and intercellularly, respectively (Montiel et al., 2021). In 12 lines, the number of pink nodules was significantly reduced at any timepoint with both rhizobial strains compared to Gifu (**Figures 2A, B**). However, nodule formation by *M. loti* was only affected in IM-53, IM-A19 and IM-D25 mutants. By contrast, the nodulation induced by IRBG74 was exclusively altered in IM-D08, IM-D11, IM-D26 and IM-N12 lines (**Figures 2A, B**, **Supplementary Figures S2A-E**). Since perturbances in the nodulation kinetics, nodule organogenesis and nitrogen fixation can negatively impact the plant growth, we measured the shoot length in the *LORE1* mutants at 6 wpi with both rhizobial strains. All the mutants tested showed a significantly shorter shoot length compared to Gifu at 6 wpi with IRBG74 (**Figure 2A**). On contrary, the aerial part was significantly affected in 11 lines inoculated with *M. loti* respect to Gifu (**Figure 2A**). These results suggest that all the mutants selected were affected in the *Lotus*-IRBG74, manifested by a delayed/reduced nodule formation or inefficient plant-growth promotion. Additionally, we observed significant differences in the root growth of several mutants at 6 wpi with IRBG74. The length was reduced in 7 lines and increased in 5 mutants (**Supplementary Figure S3**).

### *LORE1* mutants with altered root and shoot growth

**Figure 3:**
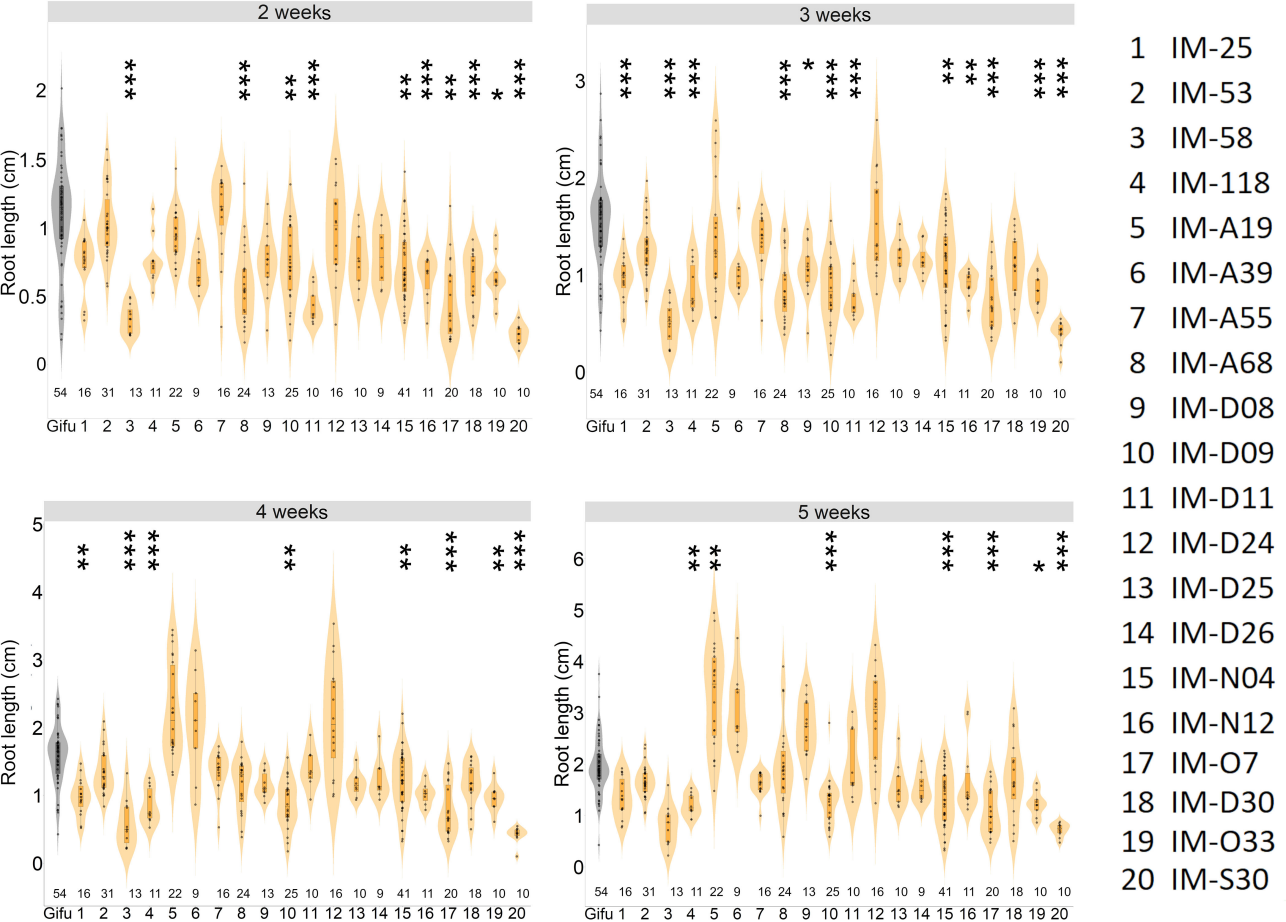
Differential root growth dynamics of *LORE1* mutants. The progression of main root growth on nitrogen-repleted medium was recorded in Gifu and *LORE1* lines at 2-5 weeks post-germination. In boxplots, the center line represents means values of 3 independent experiments; box limits, upper and lower quartiles; whiskers, 1.5× interquartile range; points represent individual data points. The number of plants tested is shown below the violin graphs. The asterisk indicates statistical significance between the *LORE1* mutants and Gifu according to Mann–Whitney U-test (*P < 0.05; **P < 0.01; ***P < 0.001).

Our study revealed that most of the selected *LORE1* mutants were perturbed in the symbiotic program triggered by IRBG74 compared to *M. loti*. Nonetheless, during our analysis we noticed that certain mutants might be also affected in root development (**Supplementary Figure S3**). We explored whether any of these mutants have additional defects in their root growth under non-symbiotic conditions, by monitoring the main root length at 2-5 weeks post-germination (wpg) on plates supplemented with 12 mM KNO_3_. In 14 lines, the root length was significantly shorter compared to Gifu plants of similar age at different timepoints. However, the length of the main root remained shorter throughout the analysis in the mutants IM-58, IM-D09, IM-N4, IM-O7, IM-O33 and IM-S30 (**Figure 3**). These results prompted us to measure the length of the aerial part in the plants grown in the above referred conditions. Although the shoot length was significantly different in 10 *LORE1* mutants compared to Gifu at 5 wpg, only in IM-58, IM-O33 and IM-S30 lines, the size was shorter (**Supplementary Figure S4**). Our data indicates that shoot and root growth is affected in several *LORE1* lines.

### Genes disrupted by *LORE1* insertions

**Figure 4:**
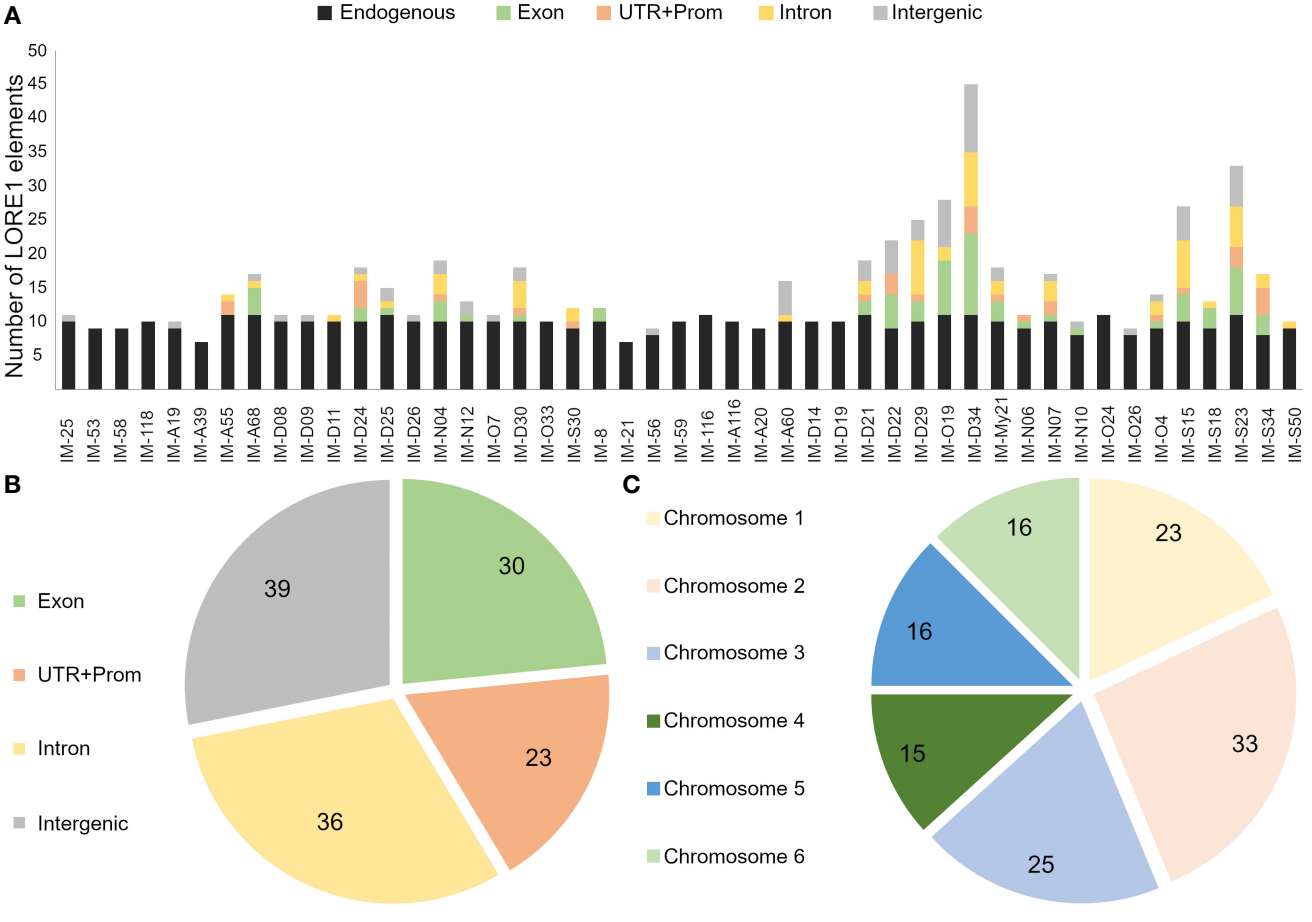
Distribution profile of retrotransposon elements in sequenced *LORE1* lines. **(A)** Number of different *LORE1* insertions detected in the genome of each mutant, including novel and endogenous elements. Venn diagrams with the number of unique *LORE1* elements found in exon, intron, UTR+Promoter, intergenic regions **(B)** and chromosomes **(C)**.

Mutants analyzed in this study, showed significant alterations in the root growth and plant-microbe associations. To get further insight into the genes associated to these phenotypes, the retrotransposon insertions were tracked in a subset of 20 mutants that were analyzed in more detail, along with 27 additional lines, randomly selected from the screening with IRBG74 (**Figure 1**, **Supplementary Figure S1**). For this purpose, we followed the FST poolit protocol that was previously developed to determine the insertion sites in the *LORE1* mutant collection (Urbanski et al., 2012; Malolepszy et al., 2016). The endogenous *LORE1* sequences and their respective position in the *Lotus* genome were successfully identified in all samples tested, confirming the effectiveness of the protocol (**Supplementary Table S1**). In 14 lines only the original *LORE1* elements were detected, however, in the remaining 33 mutants, 226 novel retrotransposon insertions were identified, 128 of them unique (**Supplementary Table S1**). These retrotransposons were distributed in introns, UTR, promoter and intergenic regions of the six chromosomes (**Figures 4A, B**). A total of 92 *Lotus* genes were disrupted in these 47 mutants and, each mutant line had in average 4.8 novel *LORE1* insertions (**Figure 4C**). Interestingly, an inspection of the expression profiles in the sequences disrupted by *LORE1* insertion in the *Lotus* database, revealed that certain genes were transcriptionally induced during symbiotic associations with rhizobia or pathogens. Therefore, these genes, interfered by *LORE1* elements, are potentially important for symbiosis (**Supplementary Figure S5**).

### Additional mutations in *LORE1* lines

**Figure 5:**
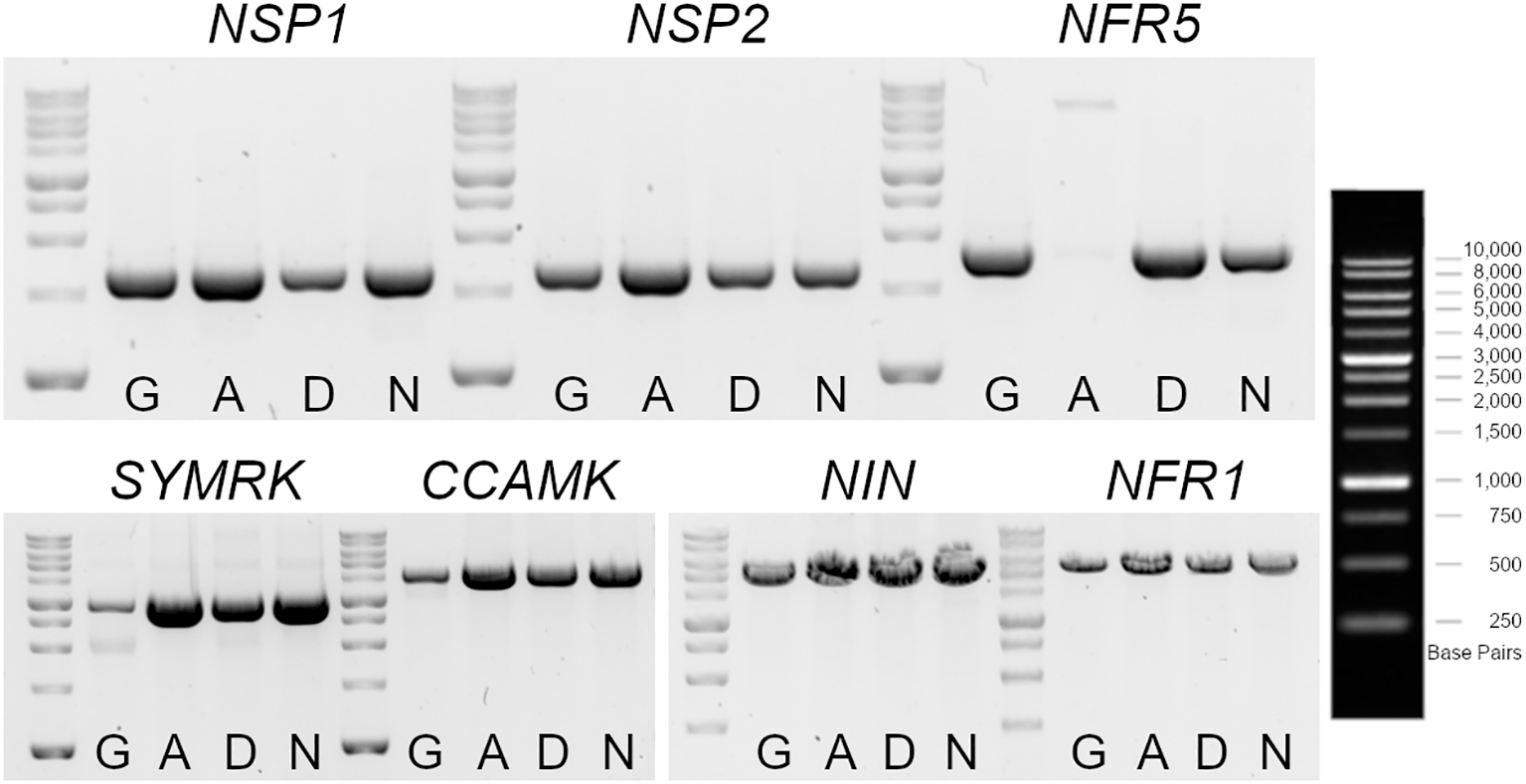
Genotyping of early symbiotic genes in *LORE1* mutants with a Nod- phenotype. Gel electrophoresis of PCR products amplified with specific oligonucleotides for *NSP1, NPS2, NFR5, SYMRK, CCAMK, NIN* and *NFR1*, using as template genomic DNA isolated from leaves of Gifu, IM-A39 (A), IM-D22 (D) and IM-N10 (N) plants. The amplicons encompass from the first to the last predicted codons for each gene. A larger fragment was obtained for the *NFR5* in the IM-A39 mutant, revealing a potential insertion within the gene.

The nodulation kinetics showed that the IM-A39 mutant was unable to develop any nodules after *M. loti* and IRBG74 inoculation (**Figure 2**). Similarly, we noticed that IM-D22 and IM-N10 lines were Nod-. The *LORE1* mapping in these plants indicates that IM-D22 contained 13 novel *LORE1* insertions, one located in the first exon of *CYCLOPS*, a crucial transcription factor for the nodulation process (Yano et al., 2008). However, the IM-A39 mutant only had endogenous *LORE1* elements and the IM-N10 line contained one intergenic and one exonic insertion, that was not located in a known early symbiotic gene (**Supplementary Table S1**). These findings suggest that non-*LORE1*-mediated mutations occurred in IM-A39 and caused the Nod- phenotype. Nod- mutants have been previously documented by different research groups, and this condition is generally caused by the loss-of-function in the early symbiotic genes *NSP1, NSP2, NFR5, SYMRK, CCAMK, NIN* and *NFR1* (Schauser et al., 1999; Radutoiu et al., 2003; Kalo et al., 2005; Heckmann et al., 2006; Yano et al., 2008; Capoen et al., 2009; Singh et al., 2014). Specific primers were designed to amplify by PCR each gene from the start to the stop predicted codon, using as template genomic DNA. The expected amplicons were obtained for all the tested genes in the different genotypes, except for *NFR5* in IM-A39. In Gifu, IM-D22 and IM-N10 mutants, a PCR product of 1.7 Kb was amplified, while in IM-A39 the amplicon size was approximately 7 Kb (**Figure 5**). Sequencing of nested PCRs products for the *NFR5* gene in the IM-A39 line revealed a *LORE2* insertion within the gene. This finding indicates that additional genomic modifications occurred in some mutants.

### Disruption of an *AUTOPHAGY9* gene affects the *Lotus*-rhizobia symbiosis

**Figure 6:**
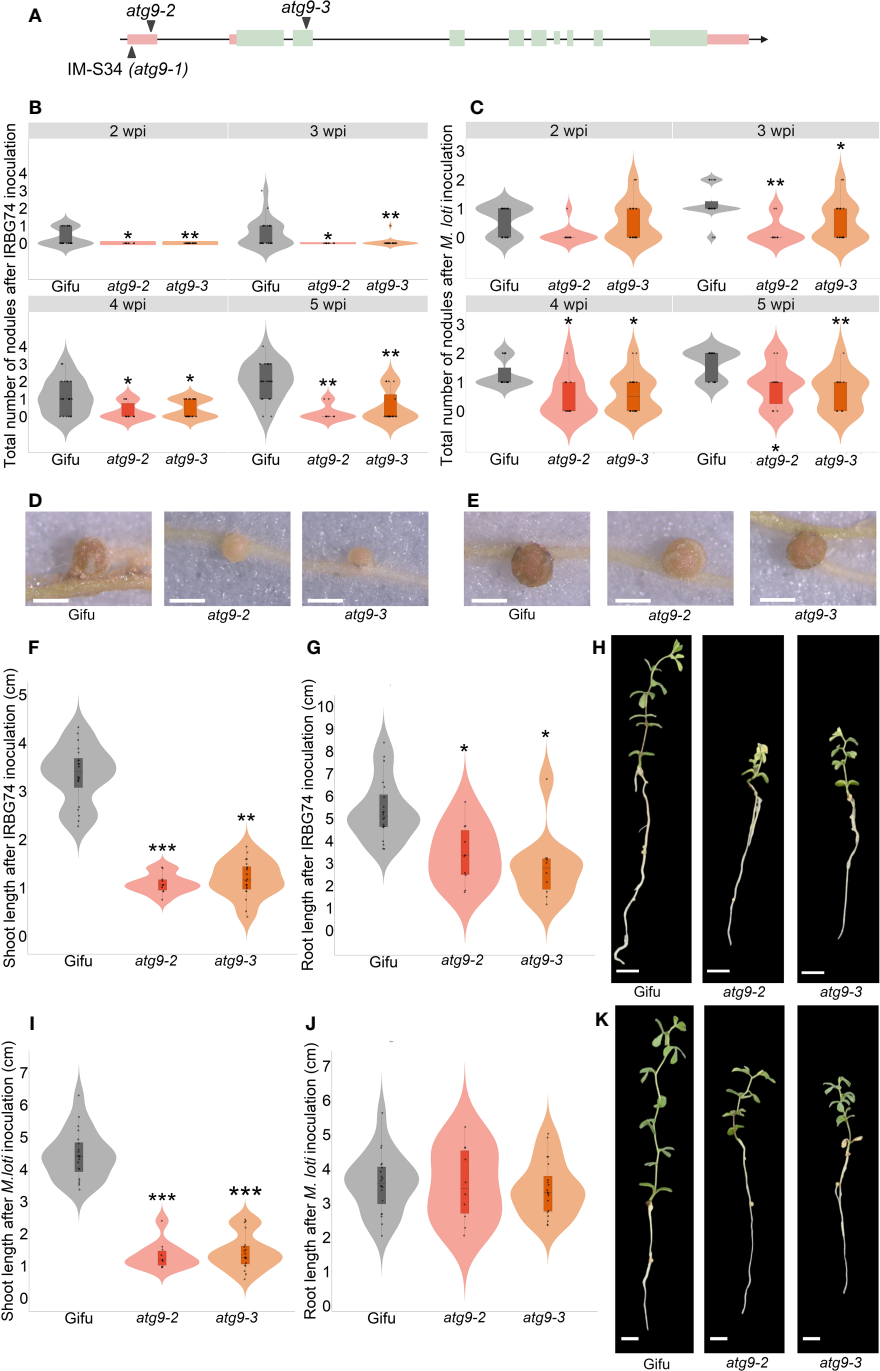
Nodulation, shoot and root phenotype of *atg9-2* and *atg9-3* mutants after inoculation with *M. loti* and IRBG74. **(A)** Scheme of the *LjATG9* gene structure. Pink rectangles, UTRs; Green rectangles, exons; Black lines, introns. Total number of nodules in Gifu (n=20), *atg9-2* (n=20) and *atg9-3* (n=10) at 2-6 wpi with IRBG74 **(B)** and *M. loti* **(C)**. Representative images of nodules formed in Gifu, *atg9-2* and *atg9-3* at 5 wpi with IRBG74 **(D)** and *M. loti* **(E)**. Scale, 1 mm. Root and shoot length measurements with representative images of Gifu, *atg9-2* and *atg9-3* at 6 wpi with IRBG74 **(F–H)** and *M. loti* **(I–K)**. Scale, 1 cm. In boxplots, the center line represents means values of 3 independent experiments; box limits, upper and lower quartiles; whiskers, 1.5× interquartile range; points represent individual data points. The asterisk indicates statistical significance between the *LORE1* mutants and Gifu according to Mann–Whitney U-test (*P < 0.05; **P < 0.01; ***P < 0.001).

Identification of insertion sites in 47 selected *LORE1* mutants provided valuable information to discover genes associated with phenotype observed. This list was compared with a collection of homozygous mutants that we have developed in our group in recent years, by genotyping *LORE1* lines previously published (Malolepszy et al., 2016). We noticed that we had two additional mutant alleles for one of the insertions found in the IM-S34 line. This mutant contained a *LORE1* insertion in the 5´UTR of the predicted *AUTOPHAGY9* gene (*ATG9*), while the additional mutant alleles were affected by a retrotransposon element in the 5´UTR (*atg9*-2) and the second exon (*atg9*-3) (**Figure 6A**). This prompted us to evaluate if the symbiotic phenotype in the IM-S34 mutant (*atg9*-1) was caused by the disruption of *ATG9*. Importantly, the IM-S34 line had a low seed production and germination rate that impeded us in performing robust nodulation kinetics with *M. loti* and IRBG74. This phenotype was probably caused by additional *LORE1* elements detected in genes highly expressed in pod and seed (**Supplementary Figure S6A**). On contrary, *ATG9* was mainly expressed in nodules at 21 dpi and induced in different root tissues at 10 dpi with *M. loti* (**Supplementary Figure S6B**; Frank et al., 2023).

The nodules numbers in the *atg9*-2 and *atg9*-3 mutants were recorded at 2-5 wpi with *M. loti* and IRBG74. In both mutants nodule development was delayed after IRBG74 inoculation, and the nodule numbers were significantly lower at 3-5 wpi with both rhizobial strains respect to Gifu (**Figures 6B, C**). Additionally, the nodules formed in these mutants were smaller and pale pink compared to Gifu (**Figures 6D, E**). The deficient nodulation in these mutants apparently had a negative impact on the growth of the plants harvested at 6 wpi with both inocula, since the shoot length was significantly shorter in *atg9*-2 and *atg9*-3 compared to Gifu (**Figures 6F, H, I, K**). Interestingly, the root length was affected in the mutants inoculated with IRBG74 but not with *M. loti* (**Figures 6G, H, J, K**). These results indicate that *LjATG9* directly or indirectly affects symbiotic program induced by *M. loti* and IRBG74.

## Discussion

### *LORE1* lines: a valuable resource for mutant screening

The screening of legume mutants disturbed in the symbiotic association with rhizobia have provided relevant information on the complex genetic network required for rhizobial infection and nodule organogenesis in model legumes (Schauser et al., 1998; Catoira et al., 2001; Kawaguchi et al., 2002; Lombardo et al., 2006; Starker et al., 2006; Pislariu et al., 2012; Domonkos et al., 2013). In this study, we conducted a large forward mutant screen, with 200,000 *LORE1* lines, scoring plants affected in the nodulation program by IRBG74. A great advantage of this approach is the relatively simple and fast insertion site sequencing methodology for identification of causative *LORE1* insertions (Urbanski et al., 2012). Compared to map-based cloning, TILLING and whole genome resequencing (Hayashi et al., 2001; Sandal et al., 2002; Sandal et al., 2006; Uchida et al., 2011; Sandal et al., 2012), the FST poolit methods is cheap, robust and fast. Different reports indicate that *LORE1* elements have a significant higher preference for genic regions, representing 55-76% of the total novel insertions (Fukai et al., 2012; Urbanski et al., 2012; Malolepszy et al., 2016). Similarly, we observed in the 47 sequenced lines, that 71% of the novel *LORE1* copies were inserted in genic regions, distributed in the different chromosomes. We detected an average of 4.8 novel insertions per plant in our mutant collection, which is comparable to the 2.7-4.7 media described in previous studies (Fukai et al., 2012; Urbanski et al., 2012; Malolepszy et al., 2016). These results reflect the efficacy of the method employed and the persistence of the retrotransposon activity in the germline of the G329-3 line.

In our selected mutant collection, 92 *Lotus* genes with *LORE1* insertions were identified. The genes compromised belong to various molecular processes, where 63 of them showed the highest expression levels under symbiotic conditions. Interestingly, we detected in this gene list two members of the cellulose synthase family, LotjaGi6g1v0183200 and LotjaGi4g1v0062500. It was recently shown that mutants affected in a *CELLULOSE SYNTHASE-LIKE D1* (*CSLD1*) gene, develop abnormal root hairs and root nodule symbiosis (Karas et al., 2021). Among the genes affected by *LORE1* elements we also found LotjaGi3g1v0273500, which encodes a predicted RhoGEF protein that shares homology with LjSPK1. In Lotus, SPK1 interacts with ROP6 to coordinate the polarized growth of ITs (Liu et al., 2020). Additionally, we also identified known symbiotic players. The Nod- phenotype of the IM-D22 mutant, correlates with the *LORE1* element detected in the coding region of *CYCLOPS*, a key transcription factor required for successful rhizobial infection and nodule organogenesis (Yano et al., 2008). However, we found that in 29% of the sequenced lines only the endogenous *LORE1* copies were detected and no additional *LORE1* insertions. This number is slightly higher to the 10-20% proportion observed in previous studies (Urbanski et al., 2012; Malolepszy et al., 2016). Therefore, the symbiotic phenotype in this set of mutants is likely mediated by non-*LORE1* mutations. This hypothesis is supported by the evidence obtained in the Nod- mutant IM-A39, where a *LORE2* element was detected in the coding region of *NFR5*, the NF receptor (Radutoiu et al., 2003). This finding is not surprising since transposition of *LORE2* elements has been documented in regenerated Gifu plants (Fukai et al., 2008). In the Nod- mutant IM-N10, large insertions/deletions were not detected in the early symbiotic genes tested, however, we cannot exclude mutations in the flanking regions of the sequences or other mutations that might impact the ORF of the genes.

### Novel LORE1 mutants interfered in the *Lotus*-IRBG74 symbiosis

In *Lotus*, the colonization by *M. loti* occurs via root-hair ITs, an intracellular infection process that has been extensively studied. However, a recent working model in *Lotus* was developed to study the intercellular infection in legumes, a largely unknown process that exists in approximately 25% of all the legume genera (Sprent, 2007). Although, the intra- and intercellular colonization in *Lotus* share some genetic components, remarkable differences have been observed among both processes (Quilbe et al., 2022). For instance, the *rinrk1* and *ern1* mutants show a more severe symbiotic phenotype with *M. loti* than with IRBG74 and by contrast, several cytoskeleton and cytokinin-related mutants are more affected in the intercellular infection (Copeland, 2021; Montiel et al., 2021). In this work, the large-scale mutant screening in boxes followed by a more stringent evaluation on plate led to the identification of 110 mutants potentially affected in the nodulation program induced by IRBG74, which colonizes *Lotus* intercellularly. A more detailed analysis in a subset of 20 mutants confirmed one of the selection criteria used in this study, since all the lines showed a significant shorter shoot length respect to Gifu after IRBG74 inoculation. Similarly, the nodule numbers were significantly reduced in 16 lines inoculated with IRBG74 in comparison to Gifu. Although the nodulation kinetics with IRBG74 was not affected in IM-53, IM-A19, IM-D25 and IM-O33 mutants, these lines showed symptoms of nitrogen starvation such as leaf chlorosis and shorter aerial part. Additionally, the nodule formation with *M. loti* was negatively impacted at different timepoints in IM-53, IM-A19 and IM-D25 lines. Only the nodulation performance of IM-O33 mutant was not affected with any rhizobial inoculum, and the compromised growth of the aerial part in this line is apparently not related to defects in mutualistic interactions with rhizobia, since the short size was also observed in nitrogen-replete conditions. The combined evidence indicates that our screening approaches showed a 95% success (19/20) to select novel mutants affected in the *Lotus*-rhizobia symbiosis, most of them perturbed in the symbiotic association of *Lotus* with IRBG74.

### Role of *LjATG9* in nodulation

Autophagy is a complex coordinated process occurring in eukaryotic organisms, to degrade and recycle cytoplasmic material, which can be induced under adverse conditions, developmental processes, or pathogenic interactions (Wang et al., 2021). We found that in the IM-S34 mutant, a *LORE1* element was inserted in the 5´UTR of the *LjATG9* gene, a putative orthologue of *Arabidopsis thaliana ATG9*, which is a component of the autophagosome (Zhuang et al., 2017). The analysis performed in additional mutant alleles, *atg9*-2 and *atg9*-3, confirmed the relevance of *ATG9* in the symbiotic association of *Lotus* with *M. loti* and IRBG74. The role of autophagy has been marginally analyzed in the legume-rhizobia symbiosis, however, it was shown in *Phaseolus vulgaris* that silencing of the autophagy-related genes *PI3K* and *BECLIN1/ATG6*, results in aborted rhizobial infection in root hairs, and reduced nodule formation (Estrada-Navarrete et al., 2016). The recycling of cellular components during rhizobial infection and nodule organogenesis is likely to occur in this complex symbiotic process, however, further research is needed to unveil the precise mechanism of autophagy during legume-rhizobia symbiosis, including a deeper characterization of *LjATG9*.

## Conclusion

*L. japonicus* is an excellent model legume with several characteristics that have facilitated the research of the legume-rhizobia symbiosis, including abundant expression data, genetic amenability and the Lotus Base, a portal that integrates transcriptomic and genomic data (Mun et al., 2016). These valuable features have been boosted with the generation of the *LORE1* mutant collection (Urbanski et al., 2012; Malolepszy et al., 2016). This resource has been widely employed by the scientific community to characterise genes-of-interest, since the retrotransposon elements can be tracked in the genome. In this study, we successfully exploited the germline activity of *LORE1a* and information in the *Lotus* base to identify novel mutants disturbed in the intercellular colonization of IRBG74 in *Lotus* roots. Importantly, the mapping of *LORE1* flanking sites revealed that uncharacterized genes transcriptionally upregulated during nodulation are likely associated to the phenotypes observed. These results reinforce the notion that certain molecular components are recruited by the plant host, depending on the infection mechanism, intracellular or intercellular. We also discovered that additional traits of interest can be tested with *LORE1* lines, such as plant development.

## Data availability statement

The original contributions presented in the study are included in the article/**Supplementary Material**. Further inquiries can be directed to the corresponding authors.

## Author contributions

IG-S: Conceptualization, Investigation, Methodology, Writing – original draft. SA: Conceptualization, Resources, Software, Supervision, Writing – review & editing. EM-M: Methodology, Writing – review & editing. MR-G: Methodology, Writing – review & editing. JG: Methodology, Writing – review & editing. NA-B: Methodology, Writing – review & editing. MS: Funding acquisition, Resources, Writing – review & editing. JS: Conceptualization, Funding acquisition, Project administration, Resources, Supervision, Visualization, Writing – original draft, Writing – review & editing. JM: Conceptualization, Funding acquisition, Investigation, Methodology, Resources, Writing – original draft.

## Supplementary material

Supplementary Table 1 Mapping of *LORE1* elements in the mutant collection.

Supplementary Table 2 List of primers used for *LORE1* genome mapping and genotyping.

## References

[B1] Bertioli D. J. Jenkins J. Clevenger J. Dudchenko O. Gao D. Seijo G.. (2019). The genome sequence of segmental allotetraploid peanut Arachis hypogaea. Nat. Genet. 51, 877–884. doi: 10.1038/s41588-019-0405-z 31043755

[B2] Broughton W. J. Dilworth M. J. (1971). Control of leghemoglobin synthesis in snake beans. Biochem. J. 125, 1075–1080. doi: 10.1042/bj1251075 5144223 PMC1178271

[B3] Capoen W. Den Herder J. Sun J. Verplancke C. De Keyser A. De Rycke R.. (2009). Calcium spiking patterns and the role of the calcium/calmodulin-dependent kinase CCaMK in lateral root base nodulation of Sesbania rostrata. Plant Cell 21, 1526–1540. doi: 10.1105/tpc.109.066233 19470588 PMC2700542

[B4] Catoira R. Timmers A. C. Maillet F. Galera C. Penmetsa R. V. Cook D.. (2001). The HCL gene of Medicago truncatula controls Rhizobium-induced root hair curling. Development 128, 1507–1518. doi: 10.1242/dev.128.9.1507 11290290

[B5] Chaintreuil C. Rivallan R. Bertioli D. J. Klopp C. Gouzy J. Courtois B.. (2016). A gene-based map of the Nod factor-independent Aeschynomene evenia sheds new light on the evolution of nodulation and legume genomes. DNA Res. 23, 365–376. doi: 10.1093/dnares/dsw020 27298380 PMC4991833

[B6] Chaudhary J. Deshmukh R. Sonah H. (2019). Mutagenesis approaches and their role in crop improvement. Plants (Basel) 8. doi: 10.3390/plants8110467 PMC6918138 31683624

[B7] Chen X. Lu Q. Liu H. Zhang J. Hong Y. Lan H.. (2019). Sequencing of Cultivated Peanut, Arachis hypogaea, Yields Insights into Genome Evolution and Oil Improvement. Mol. Plant 12, 920–934. doi: 10.1016/j.molp.2019.03.005 30902685

[B8] Copeland C. (2021). Same but different: examining the molecular mechanisms of intercellular rhizobial infection. Plant Physiol. 185, 754–756. doi: 10.1093/plphys/kiaa097 33822221 PMC8133676

[B9] Cummings S. P. Gyaneshwar P. Vinuesa P. Farruggia F. T. Andrews M. Humphry D.. (2009). Nodulation of Sesbania species by Rhizobium (Agrobacterium) strain IRBG74 and other rhizobia. Environ. Microbiol. 11, 2510–2525. doi: 10.1111/j.1462-2920.2009.01975.x 19555380 PMC7163632

[B10] Domonkos A. Horvath B. Marsh J. F. Halasz G. Ayaydin F. Oldroyd G. E.. (2013). The identification of novel loci required for appropriate nodule development in Medicago truncatula. BMC Plant Biol. 13, 157. doi: 10.1186/1471-2229-13-157 24119289 PMC3852326

[B11] Downie J. A. (2014). Legume nodulation. Curr. Biol. 24, R184–R190. doi: 10.1016/j.cub.2014.01.028 24602880

[B12] Estrada-Navarrete G. Cruz-Mireles N. Lascano R. Alvarado-Affantranger X. Hernandez-Barrera A. Barraza A.. (2016). An autophagy-related kinase is essential for the symbiotic relationship between phaseolus vulgaris and both rhizobia and arbuscular mycorrhizal fungi. Plant Cell 28, 2326–2341. doi: 10.1105/tpc.15.01012 27577790 PMC5059792

[B13] Frank M. Fechete L. I. Tedeschi F. Nadzieja M. Norgaard M. M. M. Montiel J.. (2023). Single-cell analysis identifies genes facilitating rhizobium infection in Lotus japonicus. Nat. Commun. 14, 7171. doi: 10.1038/s41467-023-42911-1 37935666 PMC10630511

[B14] Fukai E. Dobrowolska A. D. Madsen L. H. Madsen E. B. Umehara Y. Kouchi H.. (2008). Transposition of a 600 thousand-year-old LTR retrotransposon in the model legume Lotus japonicus. Plant Mol. Biol. 68, 653–663. doi: 10.1007/s11103-008-9397-2 18802778

[B15] Fukai E. Soyano T. Umehara Y. Nakayama S. Hirakawa H. Tabata S.. (2012). Establishment of a Lotus japonicus gene tagging population using the exon-targeting endogenous retrotransposon LORE1. Plant J. 69, 720–730. doi: 10.1111/j.1365-313X.2011.04826.x 22014259

[B16] Fukai E. Umehara Y. Sato S. Endo M. Kouchi H. Hayashi M.. (2010). Derepression of the plant Chromovirus LORE1 induces germline transposition in regenerated plants. PloS Genet. 6, e1000868. doi: 10.1371/journal.pgen.1000868 20221264 PMC2832683

[B17] Gossmann J. A. Markmann K. Brachmann A. Rose L. E. Parniske M. (2012). Polymorphic infection and organogenesis patterns induced by a Rhizobium leguminosarum isolate from Lotus root nodules are determined by the host genotype. New Phytol. 196, 561–573. doi: 10.1111/j.1469-8137.2012.04281.x 22950721

[B18] Handberg K. Stougaard J. (1992). Lotus japonicus, an autogamous, diploid legume species for classical and molecular genetics. Plant J. 2, 487–496. doi: 10.1111/j.1365-313X.1992.00487.x

[B19] Hayashi M. Miyahara A. Sato S. Kato T. Yoshikawa M. Taketa M.. (2001). Construction of a genetic linkage map of the model legume Lotus japonicus using an intraspecific F2 population. DNA Res. 8, 301–310. doi: 10.1093/dnares/8.6.301 11853317

[B20] Heckmann A. B. Lombardo F. Miwa H. Perry J. A. Bunnewell S. Parniske M.. (2006). Lotus japonicus nodulation requires two GRAS domain regulators, one of which is functionally conserved in a non-legume. Plant Physiol. 142, 1739–1750. doi: 10.1104/pp.106.089508 17071642 PMC1676053

[B21] Kalo P. Gleason C. Edwards A. Marsh J. Mitra R. M. Hirsch S.. (2005). Nodulation signaling in legumes requires NSP2, a member of the GRAS family of transcriptional regulators. Science 308, 1786–1789. doi: 10.1126/science.1110951 15961668

[B22] Kamal N. Mun T. Reid D. Lin J. S. Akyol T. Y. Sandal N.. (2020). Insights into the evolution of symbiosis gene copy number and distribution from a chromosome-scale Lotus japonicus Gifu genome sequence. DNA Res. 27. doi: 10.1093/dnares/dsaa015 PMC7508351 32658273

[B23] Karas B. J. Ross L. Novero M. Amyot L. Shrestha A. Inada S.. (2021). Intragenic complementation at the Lotus japonicus CELLULOSE SYNTHASE-LIKE D1 locus rescues root hair defects. Plant Physiol. 186, 2037–2050. doi: 10.1093/plphys/kiab204 34618101 PMC8331140

[B24] Karmakar K. Kundu A. Rizvi A. Z. Dubois E. Severac D. Czernic P.. (2019). Transcriptomic analysis with the progress of symbiosis in 'Crack-entry' Legume arachis hypogaea highlights its contrast with 'Infection thread' Adapted legumes. Mol. Plant Microbe Interact. 32, 271–285. doi: 10.1094/MPMI-06-18-0174-R 30109978

[B25] Kawaguchi M. Imaizumi-Anraku H. Koiwa H. Niwa S. Ikuta A. Syono K.. (2002). Root, root hair, and symbiotic mutants of the model legume Lotus japonicus. Mol. Plant Microbe Interact. 15, 17–26. doi: 10.1094/MPMI.2002.15.1.17 11843301

[B26] Liu J. Liu M. X. Qiu L. P. Xie F. (2020). SPIKE1 activates the GTPase ROP6 to guide the polarized growth of infection threads in lotus japonicus. Plant Cell 32, 3774–3791. doi: 10.1105/tpc.20.00109 33023954 PMC7721321

[B27] Lombardo F. Heckmann A. B. Miwa H. Perry J. A. Yano K. Hayashi M.. (2006). Identification of symbiotically defective mutants of Lotus japonicus affected in infection thread growth. Mol. Plant Microbe Interact. 19, 1444–1450. doi: 10.1094/MPMI-19-1444 17153928

[B28] Madsen L. H. Fukai E. Radutoiu S. Yost C. K. Sandal N. Schauser L.. (2005). LORE1, an active low-copy-number TY3-gypsy retrotransposon family in the model legume Lotus japonicus. Plant J. 44, 372–381. doi: 10.1111/j.1365-313X.2005.02534.x 16236148

[B29] Malolepszy A. Mun T. Sandal N. Gupta V. Dubin M. Urbanski D.. (2016). The LORE1 insertion mutant resource. Plant J. 88, 306–317. doi: 10.1111/tpj.13243 27322352

[B30] Mikkers H. Allen J. Knipscheer P. Romeijn L. Hart A. Vink E.. (2002). High-throughput retroviral tagging to identify components of specific signaling pathways in cancer. Nat. Genet. 32, 153–159. doi: 10.1038/ng950 12185366

[B31] Montiel J. Garcia-Soto I. James E. K. Reid D. Cardenas L. Napsucialy-Mendivil S.. (2023). Aromatic amino acid biosynthesis impacts root hair development and symbiotic associations in Lotus japonicus. Plant Physiol. 193, 1508–1526. doi: 10.1093/plphys/kiad398 37427869 PMC10517252

[B32] Montiel J. Reid D. Gronbaek T. H. Benfeldt C. M. James E. K. Ott T.. (2021). Distinct signaling routes mediate intercellular and intracellular rhizobial infection in Lotus japonicus. Plant Physiol. 185, 1131–1147. doi: 10.1093/plphys/kiaa049 33793909 PMC8133683

[B33] Mun T. Bachmann A. Gupta V. Stougaard J. Andersen S. U. (2016). Lotus Base: An integrated information portal for the model legume Lotus japonicus. Sci. Rep. 6, 39447. doi: 10.1038/srep39447 28008948 PMC5180183

[B34] Peng Z. Liu F. Wang L. Zhou H. Paudel D. Tan L.. (2017). Transcriptome profiles reveal gene regulation of peanut (Arachis hypogaea L.) nodulation. Sci. Rep. 7, 40066. doi: 10.1038/srep40066 28059169 PMC5216375

[B35] Pislariu C. I. Murray J. D. Wen J. Cosson V. Muni R. R. Wang M.. (2012). A Medicago truncatula tobacco retrotransposon insertion mutant collection with defects in nodule development and symbiotic nitrogen fixation. Plant Physiol. 159, 1686–1699. doi: 10.1104/pp.112.197061 22679222 PMC3425206

[B36] Quilbe J. Lamy L. Brottier L. Leleux P. Fardoux J. Rivallan R.. (2021). Genetics of nodulation in Aeschynomene evenia uncovers mechanisms of the rhizobium-legume symbiosis. Nat. Commun. 12, 829. doi: 10.1038/s41467-021-21094-7 33547303 PMC7864950

[B37] Quilbe J. Montiel J. Arrighi J. F. Stougaard J. (2022). Molecular mechanisms of intercellular rhizobial infection: novel findings of an ancient process. Front. Plant Sci. 13, 922982. doi: 10.3389/fpls.2022.922982 35812902 PMC9260380

[B38] Radutoiu S. Madsen L. H. Madsen E. B. Felle H. H. Umehara Y. Gronlund M.. (2003). Plant recognition of symbiotic bacteria requires two LysM receptor-like kinases. Nature 425, 585–592. doi: 10.1038/nature02039 14534578

[B39] Raul B. Bhattacharjee O. Ghosh A. Upadhyay P. Tembhare K. Singh A.. (2022). Microscopic and transcriptomic analyses of dalbergoid legume peanut reveal a divergent evolution leading to nod-factor-dependent epidermal crack-entry and terminal bacteroid differentiation. Mol. Plant Microbe Interact. 35, 131–145. doi: 10.1094/MPMI-05-21-0122-R 34689599

[B40] Rogers S. O. Bendich A. J. (1985). Extraction of DNA from milligram amounts of fresh, herbarium and mummified plant tissues. Plant Mol. Biol. 5, 69–76. doi: 10.1007/BF00020088 24306565

[B41] Roy S. Liu W. Nandety R. S. Crook A. Mysore K. S. Pislariu C. I.. (2020). Celebrating 20 years of genetic discoveries in legume nodulation and symbiotic nitrogen fixation. Plant Cell 32, 15–41. doi: 10.1105/tpc.19.00279 31649123 PMC6961631

[B42] Sandal N. Jin H. Rodriguez-Navarro D. N. Temprano F. Cvitanich C. Brachmann A.. (2012). A set of Lotus japonicus Gifu x Lotus burttii recombinant inbred lines facilitates map-based cloning and QTL mapping. DNA Res. 19, 317–323. doi: 10.1093/dnares/dss014 22619310 PMC3415293

[B43] Sandal N. Krusell L. Radutoiu S. Olbryt M. Pedrosa A. Stracke S.. (2002). A genetic linkage map of the model legume Lotus japonicus and strategies for fast mapping of new loci. Genetics 161, 1673–1683. doi: 10.1093/genetics/161.4.1673 12196410 PMC1462218

[B44] Sandal N. Petersen T. R. Murray J. Umehara Y. Karas B. Yano K.. (2006). Genetics of symbiosis in Lotus japonicus: recombinant inbred lines, comparative genetic maps, and map position of 35 symbiotic loci. Mol. Plant Microbe Interact. 19, 80–91. doi: 10.1094/MPMI-19-0080 16404956

[B45] Schauser L. Handberg K. Sandal N. Stiller J. Thykjaer T. Pajuelo E.. (1998). Symbiotic mutants deficient in nodule establishment identified after T-DNA transformation of Lotus japonicus. Mol. Gen. Genet. 259, 414–423. doi: 10.1007/s004380050831 9790598

[B46] Schauser L. Roussis A. Stiller J. Stougaard J. (1999). A plant regulator controlling development of symbiotic root nodules. Nature 402, 191–195. doi: 10.1038/46058 10647012

[B47] Singh S. Katzer K. Lambert J. Cerri M. Parniske M. (2014). CYCLOPS, a DNA-binding transcriptional activator, orchestrates symbiotic root nodule development. Cell Host Microbe 15, 139–152. doi: 10.1016/j.chom.2014.01.011 24528861

[B48] Sprent J. I. (2007). Evolving ideas of legume evolution and diversity: a taxonomic perspective on the occurrence of nodulation. New Phytol. 174, 11–25. doi: 10.1111/j.1469-8137.2007.02015.x 17335493

[B49] Starker C. G. Parra-Colmenares A. L. Smith L. Mitra R. M. Long S. R. (2006). Nitrogen fixation mutants of Medicago truncatula fail to support plant and bacterial symbiotic gene expression. Plant Physiol. 140, 671–680. doi: 10.1104/pp.105.072132 16407449 PMC1361333

[B50] Tadege M. Wen J. He J. Tu H. Kwak Y. Eschstruth A.. (2008). Large-scale insertional mutagenesis using the Tnt1 retrotransposon in the model legume Medicago truncatula. Plant J. 54, 335–347. doi: 10.1111/j.1365-313X.2008.03418.x 18208518

[B51] Uchida N. Sakamoto T. Kurata T. Tasaka M. (2011). Identification of EMS-induced causal mutations in a non-reference Arabidopsis thaliana accession by whole genome sequencing. Plant Cell Physiol. 52, 716–722. doi: 10.1093/pcp/pcr029 21398646

[B52] Urbanski D. F. Malolepszy A. Stougaard J. Andersen S. U. (2012). Genome-wide LORE1 retrotransposon mutagenesis and high-throughput insertion detection in Lotus japonicus. Plant J. 69, 731–741. doi: 10.1111/j.1365-313X.2011.04827.x 22014280

[B53] Wang P. Wang T. Han J. Li M. Zhao Y. Su T.. (2021). Plant autophagy: an intricate process controlled by various signaling pathways. Front. Plant Sci. 12, 754982. doi: 10.3389/fpls.2021.754982 34630498 PMC8495024

[B54] Yano K. Yoshida S. Muller J. Singh S. Banba M. Vickers K.. (2008). CYCLOPS, a mediator of symbiotic intracellular accommodation. Proc. Natl. Acad. Sci. U.S.A. 105, 20540–20545. doi: 10.1073/pnas.0806858105 19074278 PMC2629324

[B55] Zarrabian M. Montiel J. Sandal N. Ferguson S. Jin H. Lin Y. Y.. (2022). A promiscuity locus confers Lotus burttii nodulation with rhizobia from five different genera. Mol. Plant Microbe Interact 11, 1006–1017. doi: 10.1094/MPMI-06-22-0124-R 35852471

[B56] Zhuang X. Chung K. P. Cui Y. Lin W. Gao C. Kang B. H.. (2017). ATG9 regulates autophagosome progression from the endoplasmic reticulum in Arabidopsis. Proc. Natl. Acad. Sci. U.S.A. 114, E426–E435. doi: 10.1073/pnas.1616299114 28053229 PMC5255614

