# Supplementary material for: A collection of novel Lotus japonicus LORE1 mutants perturbed in the nodulation program induced by the Agrobacterium pusense strain IRBG74
Source: Front Plant Sci. 2024 Jan 5;14:1326766. doi: 10.3389/fpls.2023.1326766 (PMC10796720; doi:10.3389/fpls.2023.1326766)
Supplement: Supplementary file 1 [file DataSheet_1.pdf]

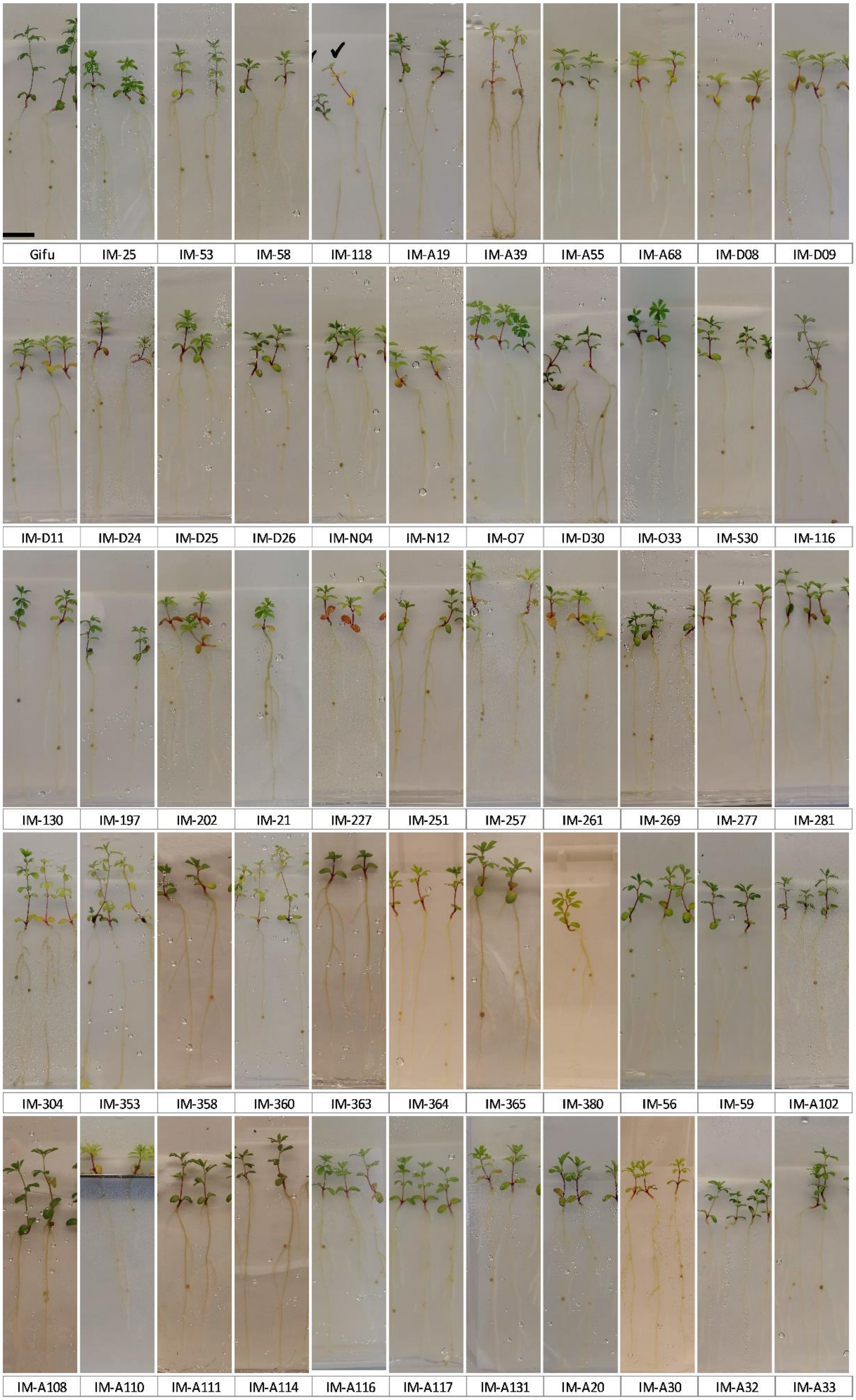
Gifu
IM-25
IM-53
IM-58
IM-118
IM-A19
IM-A39
IM-A55
IM-A68
IM-D08
IM-D09
IM-D11
IM-D24
IM-D25
IM-D26
IM-N04
IM-N12
IM-O7
IM-D30
IM-O33
IM-S30
IM-116
IM-130
IM-197
IM-202
IM-21
IM-227
IM-251
IM-257
IM-261
IM-269
IM-277
IM-281
IM-304
IM-353
IM-358
IM-360
IM-363
IM-364
IM-365
IM-380
IM-56
IM-59
IM-A102
IM-A108
IM-A110
IM-A111
IM-A114
IM-A116
IM-A117
IM-A131
IM-A20
IM-A30
IM-A32
IM-A33

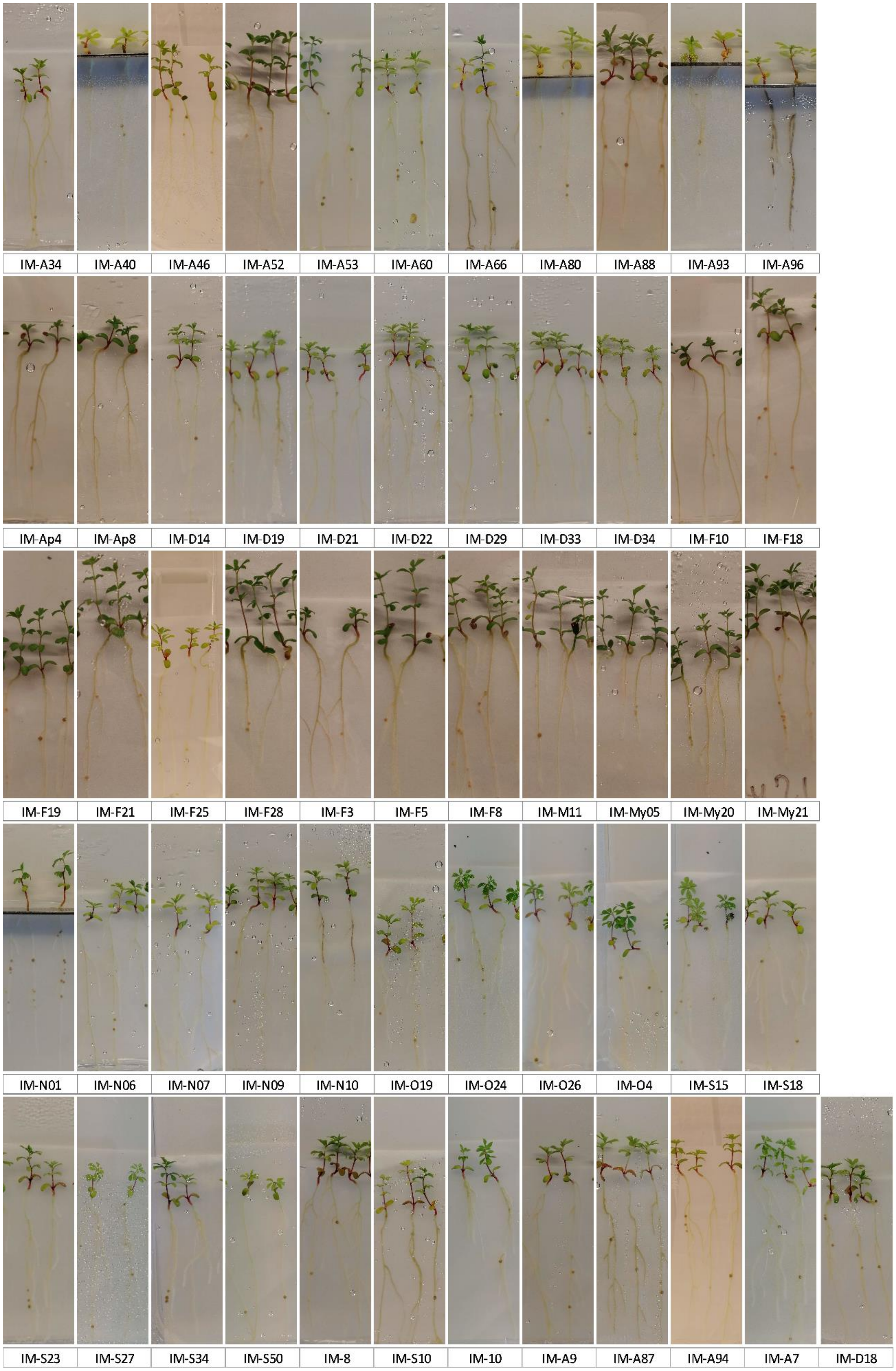
IM-A34
IM-A40
IM-A46
IM-A52
IM-A53
IM-A60
IM-A66
IM-A80
IM-A88
IM-A93
IM-A96
IM-Ap4
IM-Ap8
IM-D14
IM-D19
IM-D21
IM-D22
IM-D29
IM-D33
IM-D34
IM-F10
IM-F18
IM-F19
IM-F21
IM-F25
IM-F28
IM-F3
IM-F5
IM-F8
IM-M11
IM-My05
IM-My20
IM-My21
IM-N01
IM-N06
IM-N07
IM-N09
IM-N10
IM-O19
IM-O24
IM-O26
IM-O4
IM-S15
IM-S18
IM-S23
IM-S27
IM-S34
IM-S50
IM-8
IM-S10
IM-10
IM-A9
IM-A87
IM-A94
IM-A7
IM-D18

Figure S1. Selected mutants with symbiotic phenotype. Representative images of the *LORE1* mutants selected from the screening showing a Nod-, Fix- and hypernodulation phenotypes at 6 wpi with IRBG74. Scale, 1 cm. Images obtained at the same magnification.

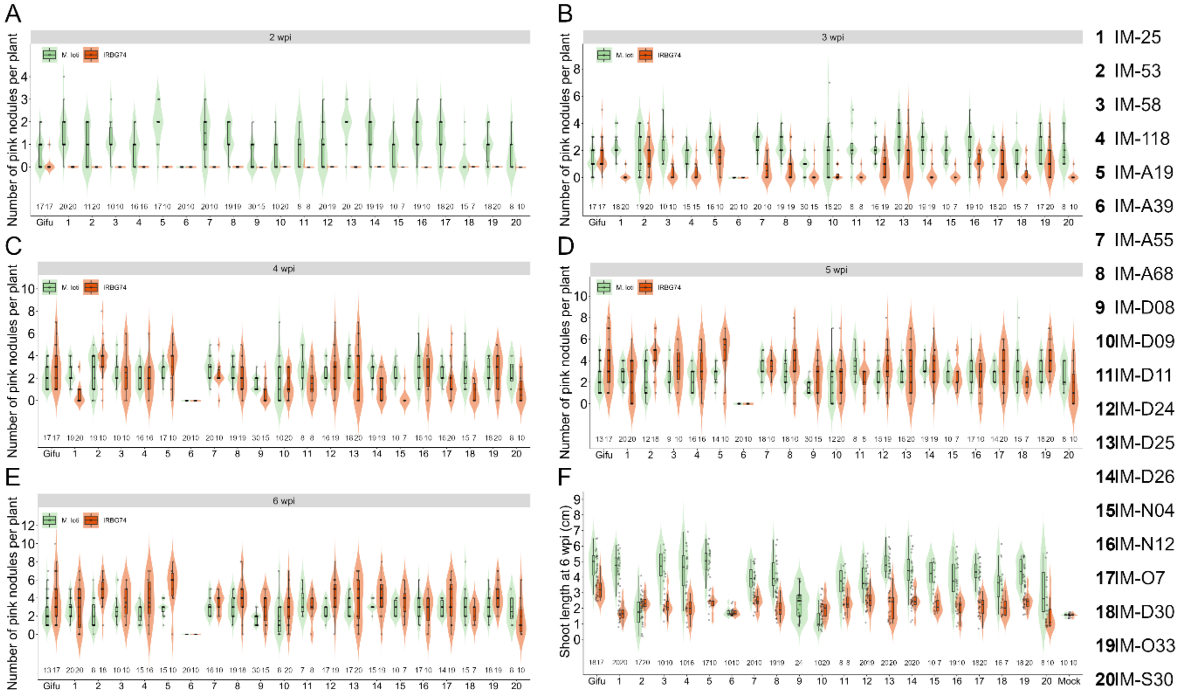

Figure S2. Delayed and reduced nodulation in novel *LORE1* mutants. **A-E**, Number of pink nodules in Gifu and 20 *LORE1* mutants at 2-6 wpi with *M. loti* and IRBG74. Violin boxplots: center line, median; box limits, upper and lower quartiles; whiskers, 1.5× interquartile range; points, individual data points. **F**, Shoot length in Gifu and 20 *LORE1* mutant at 6 wpi with *M. loti* and IRBG74. The number of plants tested is shown below the violin graphs.

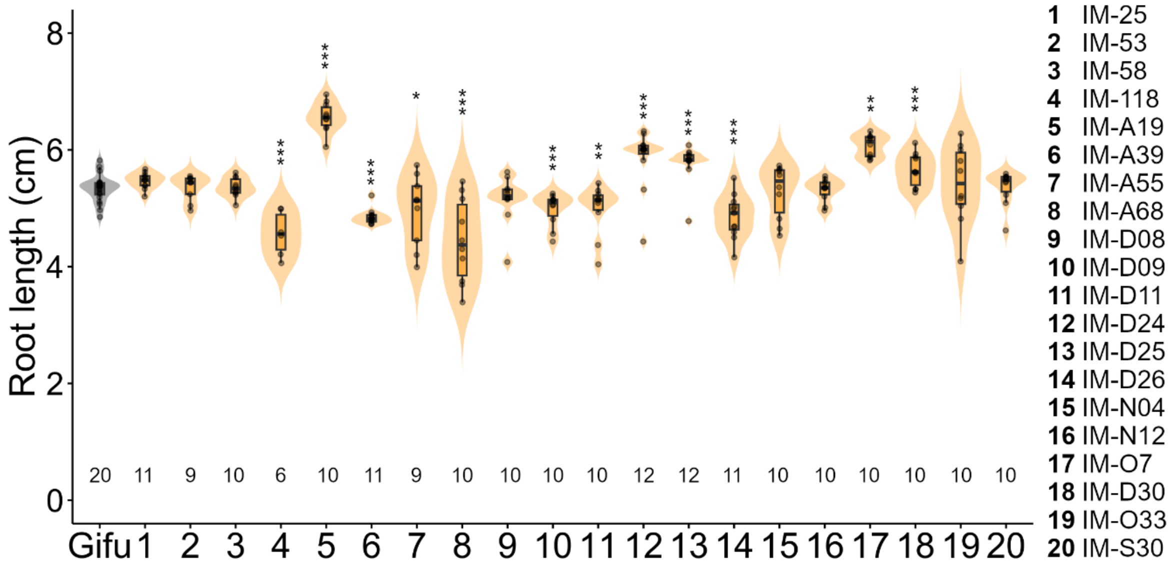

Figure S3. Root length of 20 *LORE1* lines inoculated with IRBG74. Violin dot plots showing the root length at 6 wpi with IRBG74. Center line, median; box limits, upper and lower quartiles; whiskers, 1.5× interquartile range; points, individual data points. The number of tested plants is shown below the violin graphs. * P<0.05; **P<0.01; and *** P<0.001 Mann–Whitney U-test.

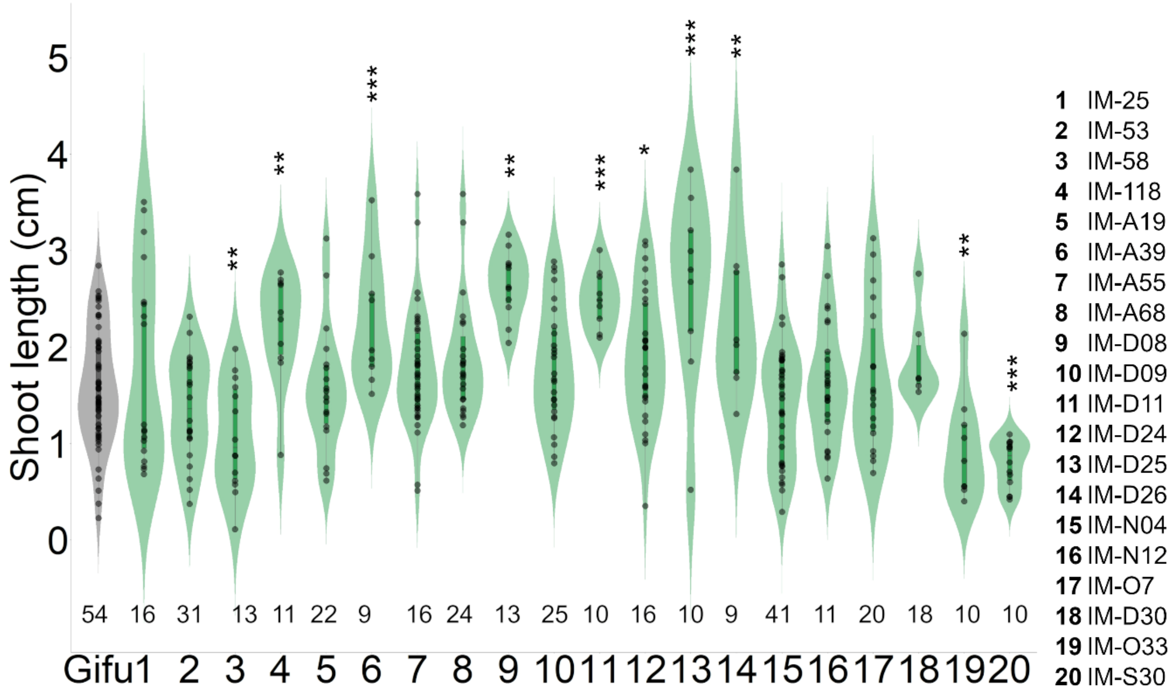

Figure S4. Shoot length of 20 LORE1 lines in nitrogen-repleted conditions. Violin dot plots showing the shoot length at 5 wpg of the selected mutants grown on plates with optimal nitrogen supply. Center line, median; box limits, upper and lower quartiles; whiskers, 1.5× interquartile range; points, individual data points. The number of tested plants is shown below the violin graphs. * $P<0.05$; ** $P<0.01$; and *** $P<0.001$ Mann–Whitney *U*-test.

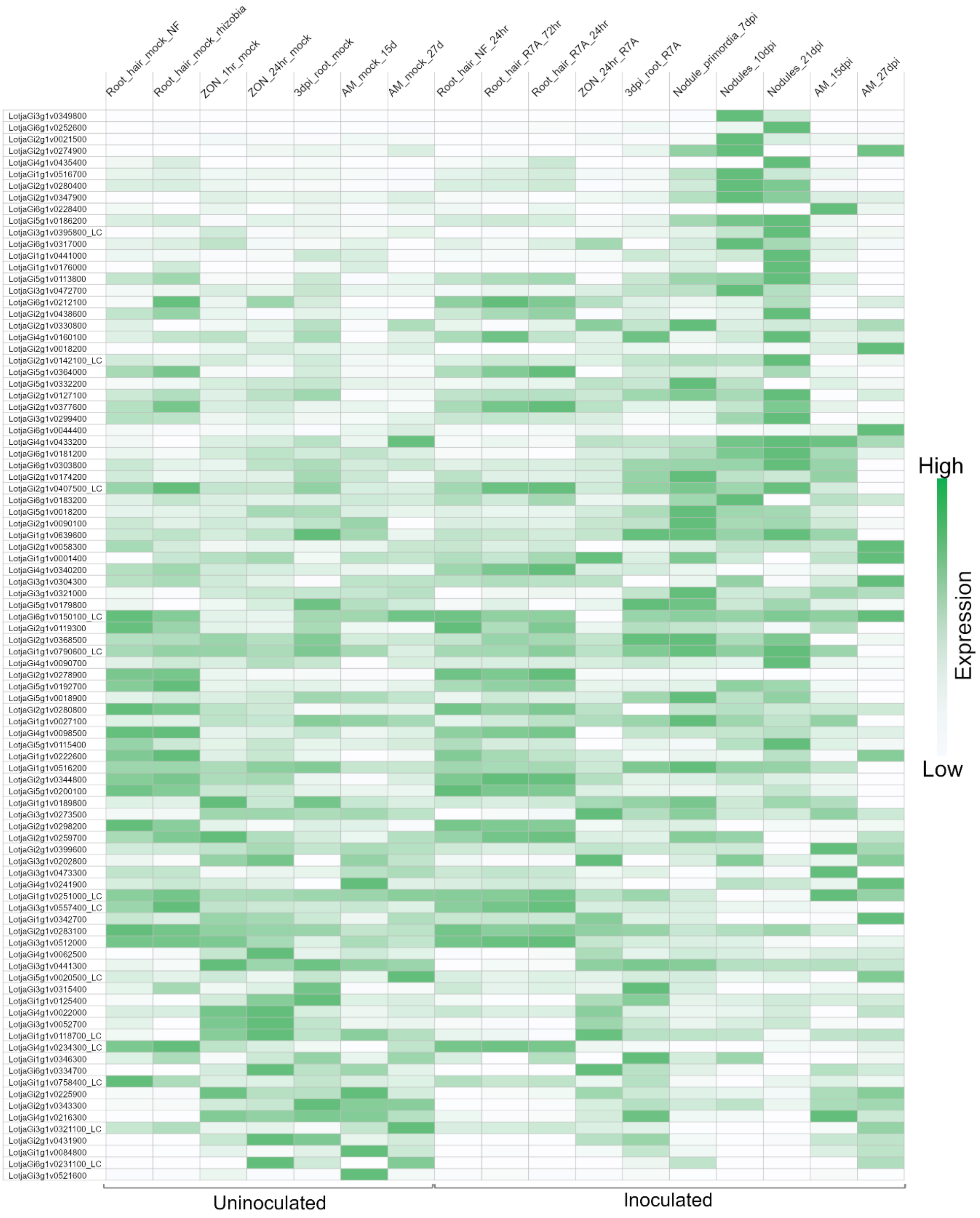

Figure S5. Heatmap expression of genes affected by *LORE1* insertions in the mutant collection. Expression pattern of the genes that showed *LORE1* insertions in 47 mutants selected in this study. The data was extracted from the Lotus Expression Atlas https://lotus.au.dk/expat/.

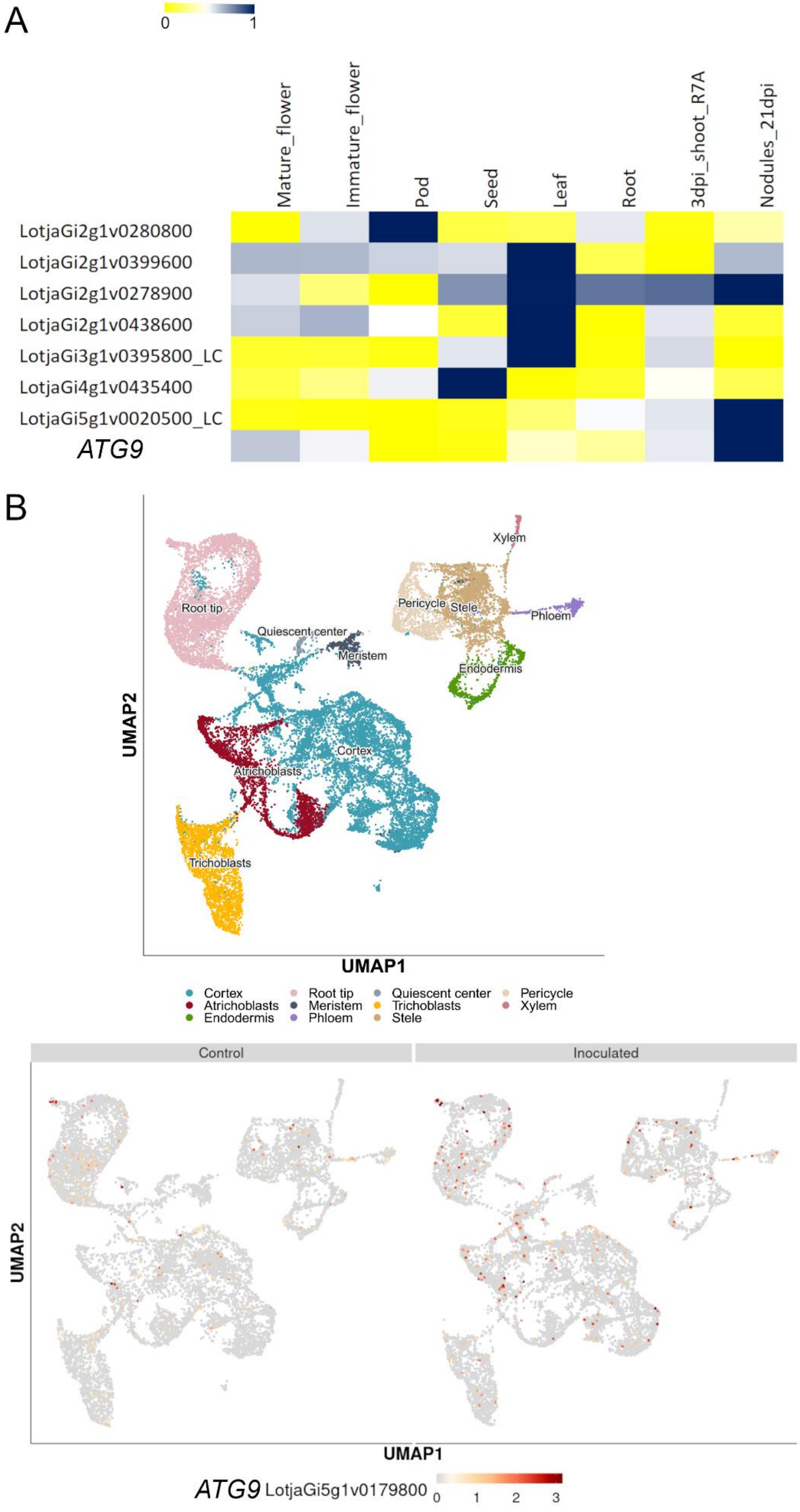
A
0
1
Mature_flower
Immature_flower
Pod
Seed
Leaf
Root
3dpi_shoot_R7A
Nodules_21dpi
LotjaGi2g1v0280800
LotjaGi2g1v0399600
LotjaGi2g1v0278900
LotjaGi2g1v0438600
LotjaGi3g1v0395800_LC
LotjaGi4g1v0435400
LotjaGi5g1v0020500_LC
ATG9
B
Xylem
Pericycle
Stele
Phloem
Root tip
Quiescent center
Meristem
Endodermis
Cortex
Atrichoblasts
Trichoblasts
UMAP2
UMAP1
Cortex
Atrichoblasts
Endodermis
Root tip
Meristem
Phloem
Quiescent center
Trichoblasts
Stele
Pericycle
Xylem
Control
Inoculated
UMAP2
UMAP1
ATG9 LotjaGi5g1v0179800
0
1
2
3

Figure S6. Expression profile of sequences disrupted by *LORE1* elements. **A**, Heatmap of normalized expression of genes affected by *LORE1* insertions in the IM-S34 mutant. Data extracted from the Lotus Expression Atlas https://lotus.au.dk/expat/. **B**, UMAPs of *ATG9* expression in different root tissues at 10 dpi with *M. loti* and mock-treated (control). Collected from *Lotus japonicus* root single-cell database (Frank et al., 2023).
